# Electrochemical Performance of LiTa_2_PO_8_-Based Succinonitrile Composite Solid Electrolyte without Sintering Process

**DOI:** 10.3390/ma17194882

**Published:** 2024-10-04

**Authors:** Nayoung Kim, Wongyeong Park, Hyeonjin Kim, Seog-young Yoon

**Affiliations:** 1 Department of Materials Science and Engineering, Pusan National University, Busan 46241, Republic of Korea; ny3726@pusan.ac.kr (N.K.); gtjsdk5542@pusan.ac.kr (W.P.); 2 A1 Project, Corporate R&D Institute, Samsung Electro-Mechanics Co., Ltd., Suwon 16674, Republic of Korea

**Keywords:** succinonitrile, interface, non-sintering process, LiTa_2_PO_8_, composite solid electrolyte

## Abstract

Solid-state batteries (SSBs) have been widely studied as next-generation lithium-ion batteries (LiBs) for many electronic devices due to their high energy density, stability, nonflammability, and chemical stability compared to LiBs which consist of liquid electrolytes. However, solid electrolytes exhibit poor electrochemical characteristics due to their interfacial properties, and the sintering process, which necessitates high temperatures, is an obstacle to the commercialization of SSBs. Hence, the aim of this study was to improve the interfacial properties of the lithium tantalum phosphate (LTPO) solid electrolyte by adding succinonitrile (SN) on the interface of the LTPO particle to enhance ionic conductivity without the sintering process. Electrochemical impedance spectroscopy (EIS), the Li symmetric cell test, and the galvanostatic cycle test were performed to verify the performance of the SN-containing LTPO composite electrolyte. The LTPO composite solid electrolyte exhibited a high ionic conductivity of 1.93 × 10^−4^ S/cm at room temperature (RT) compared to the conventional LTPO. Also, it showed good cycle stability, and low interfacial resistance with Li metal, ensuring electrochemical stability. On the basis of our experimental results, the performance of solid electrolytes could be improved by adding SN and lithium salt. In addition, the SN can be used to fabricate the solid electrolytes without the sintering process at high temperatures.

## 1. Introduction

Recently, LIBs have become very important as low-cost, environmentally sustainable, and high-efficiency energy storage systems, because of the exhaustion of fossil fuels and the growing interest in sustainable energy [1,2,3,4,5,6]. LIBs are essential elements in small-sized electronic devices as well as in mid- to large-sized energy conversion and storage systems such as electric vehicles [4,5]. LIBs composed of liquid electrolytes, which are currently the most widely commercialized, have excellent electrochemical properties in that they have high energy density and ionic conductivity. However, LIBs composed of liquid electrolytes use organic solvents that cause significant safety concerns, including due to their flammability and thermal instability, which can potentially lead to explosion problems [6,7,8,9]. Hence, research on next-generation LIBs, namely SSBs composed of a solid electrolyte (SE), has been suggested for the designing of safe LIBs [10,11,12,13,14]. SSBs exhibit high energy density and excellent thermal stability, effectively eliminating safety concerns [10,11,12]. Nevertheless, the majority of SSBs exhibit a lower ionic conductivity than those employing liquid electrolytes, which prevents the commercialization of SEs [1,10]. Additionally, the conventional ceramic sintering process has the problem of requiring high temperature and time, which results in the consumption of a considerable amount of energy that is not essential. To solve this issue, many studies attempt to enhance performance by using specific polymers or other compounds without high-temperature sintering [15,16,17]. However, they inevitably require a sintering process such as low-temperature sintering. Therefore, some fundamental requirements for SEs are necessary: high ionic conductivity of at least 10^−4^ S/cm with low electronic conductivity, excellent electrochemical stability, and elimination of high-temperature sintering processes that use a lot of energy [18].

In the case of SEs, there are oxide-based inorganic SEs and sulfide-based inorganic SEs, consisting of oxide or sulfide ionic conductors, and composite SEs, which combine organic and inorganic SEs [19,20,21,22,23]. Among the oxide-based SEs, LiTa_2_PO_8_ (LTPO) is one of the most widely studied materials due to its high ionic conductivity. LTPO fabricated by a solid-state reaction has an ionic conductivity of 2.5 × 10^−4^ S/cm at room temperature [24,25,26,27]. To advance the electrochemical performance of LTPO, some research has been conducted, including in doping and interfacial performance improvement [27,28,29,30,31,32]. One method for enhancing the interfacial performance of oxide-based solid electrolytes is to incorporate SN [31,33,34]. However, there are no studies that have demonstrated an improvement in the ionic conductivity of LTPO upon the addition of succinonitrile (SN). SN is one of the non-ionic and highly polar solid organic plastic materials that lithium salt dissolves well in. SN improves the thermal stability and ionic conductivity of electrolytes when complexed with lithium salt [34,35]. When the SN and lithium salt are blended with the SE, the strong polarity of SN molecules facilitates the dissolution of lithium salt, and the interactions between nitrile groups (-C≡N) and Li^+^ enhance the ionic conductivity of the SE [34,35].

In this study, LTPO ceramic matrix-based composite solid electrolyte was fabricated without the high-temperature sintering process. To improve the electrochemical performance of the LTPO solid electrolyte, SN and lithium salt were introduced during the synthesizing of the LTPO composite powder. The influence of the SN and lithium salt on the microstructure and electrochemical properties of the LTPO composite pellets with different contents of LTPO–SN–LiTFSI was investigated. Furthermore, the electrochemical performance test was conducted to estimate the electrochemical stability of LTPO composite pellets.

## 2. Materials and Methods

### 2.1. Synthesis of the LTPO and LTPO Composite Powder

LiTa_2_PO_8_ powders were synthesized with a solid-state method. Lithium carbonate Li_2_CO_3_ (Junsei, London, UK, 99.00%), Tantalum (V) oxide Ta_2_O_5_ (Thermo-Scientific, Waltham, MA, USA, 99.85%), and Ammonium phosphate monobasic NH_4_H_2_PO_4_ (Duksan, Yesan, Republic of Korea, 98.00%) were used as precursors. The precursors were mixed using a stoichiometric ratio of Li:Ta:P = 1:2:1 in an alumina mortar. The powder prepared above was calcined at 600 °C for 6 h through a two-step heating process which was carried out at 800 °C for 1 h and then at 1050 °C for 12 h. The obtained powders were roughly crushed and then milled with a planetary ball mill process at 125 rpm for 24 h with 70 g of 5 mm ZrO_2_ balls and ethanol in a ZrO_2_ jar to refine the particle size. After the milling process, the average size of LTPO particles was around 450 nm.

For preparing the LTPO composite powders, succinonitrile (Sigma-Aldrich, St. Louis, MO, USA, 99.00%) was used in the form of an SN solution using N,N-Dimethylformamide (99.80%, Daejung Co., Ltd., Busan, Republic of Korea). The LTPO powder, LiTFSI (Sigma-Aldrich, 99.90%), and SN solution were stirred in DMF and dried at 100 °C. The ratio of SN to LiTFSI was synthesized at a 1:1 ratio, and the composition of the samples was as follows: (100 − x) wt% LTPO:x wt% SN + LiTFSI, with values of x equal to 0, 2.5, 5, 7.5, 10, 12.5, and 15.

### 2.2. Fabrication of LTPO Composite Pellets

**Figure 1:**
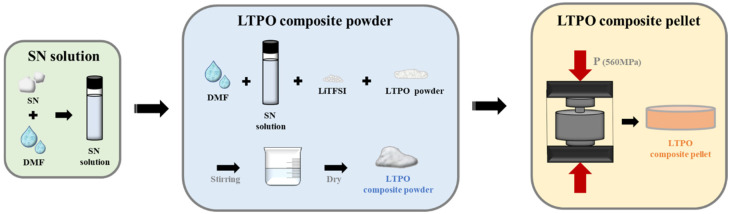
The schematic diagram of the experimental procedure for preparing LTPO composite samples.

The overall fabrication process of LTPO composite samples is illustrated in Figure 1. The obtained LTPO composite powders were compressed into pellets with dimensions of 10 mm in 560 MPa at RT. The microstructure and electrochemical properties of the LTPO composite samples were represented as the different LTPO–SN ratios to investigate the effect of the contents of SN and LiTFSI, and their notations are displayed in Table 1.

### 2.3. Characterization

The phases and crystal structures of the synthesized LTPO and LTPO composite powders were investigated via X-ray diffraction (XRD, UltimaIV, Rigaku, Tokyo, Japan) using Cu Kα radiation (l = 1.5428 A, 40 kV, 40 mA) for a 2θ range from 10° to 60°, with a step size and duration time of 0.02 and 70s, respectively. The morphologies of the synthesized LTPO composite pellets were observed using a field-emission scanning electron microscope (FE-SEM, MIRA 3, TESCAN, Dortmund, Germany). To measure the relative densities of the pellets, we compared the measured density to the theoretical density (LTPO: 5.848 g/cm^3^, SN: 1.77 g/cm^3^). To identify the SN-contained area at the LTPO interface and physical or chemical interactions between SN and LTPO, a field-emission transmission electron microscope (FE-TEM, JEOL/JEM-F200, Tokyo, Japan) was used. The ionic conductivities of the samples were measured using electrochemical impedance spectroscopy (EIS, VSP-300, Bio-Logic, Seyssinet-Pariset, France). The Z view program, which performs fitting calculations, was used to measure the ionic conductivity. To measure the electronic conductivities of the samples, a potentiostat (VersaSTAT4, AMETEK, Berwyn, PA, USA) was used at RT and 1 V of an applied voltage until a steady-state current occurred. The activation energies of the samples were measured over a temperature range of 25–85 °C. The electronic conductivity and activation energy of the samples are calculated from the fitting results and the two formulas as shown below

σ = L/(R∙S),(1)

σT = Aexp(−E_a_/kT). (2)

where σ represents the conductivity, L is the thickness of the sample, R is the value of impedance from the fitted equivalent circuit, S is the surface area of the sample, E_a_ is the activation energy of the sample, and A, k, and T are the pre-exponential factor, Boltzmann’s constant and absolute temperature, respectively [29].

## 3. Results and Discussion

**Figure 2:**
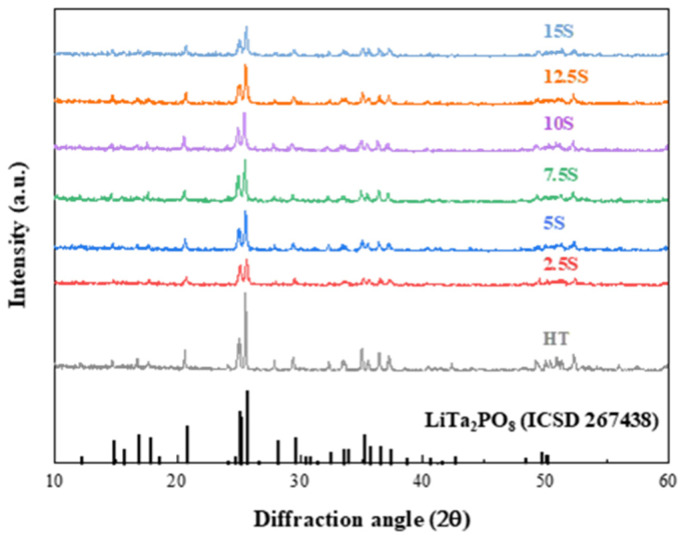
X-ray diffractions of the LTPO composite samples with different SN to LiTFSI ratios.

Figure 2 shows the X-ray diffraction patterns of the LTPO composite pellets with different SN and LiTFSI ratios. The diffraction peaks of overall LTPO composite pellets showed only the characteristic LTPO monoclinic phase ICSD 267438 regardless of the amount of SN and LiTFSI, which is well matched with other studies [26,27,29]. It was assumed that the phase of LTPO was not affected by the SN and LiTFSI solutions during the preparation process. This implies that the LTPO composite pellets as solid electrolyte were successfully synthesized through the process shown in Figure 1. In addition, the intensity of the characteristic peak corresponding to the LTPO phase was slightly decreased and widened with the addition of SN and LiTFSI. It could be assumed that the plastic material SN attenuated the crystallinity of the LTPO phase.

**Figure 3:**
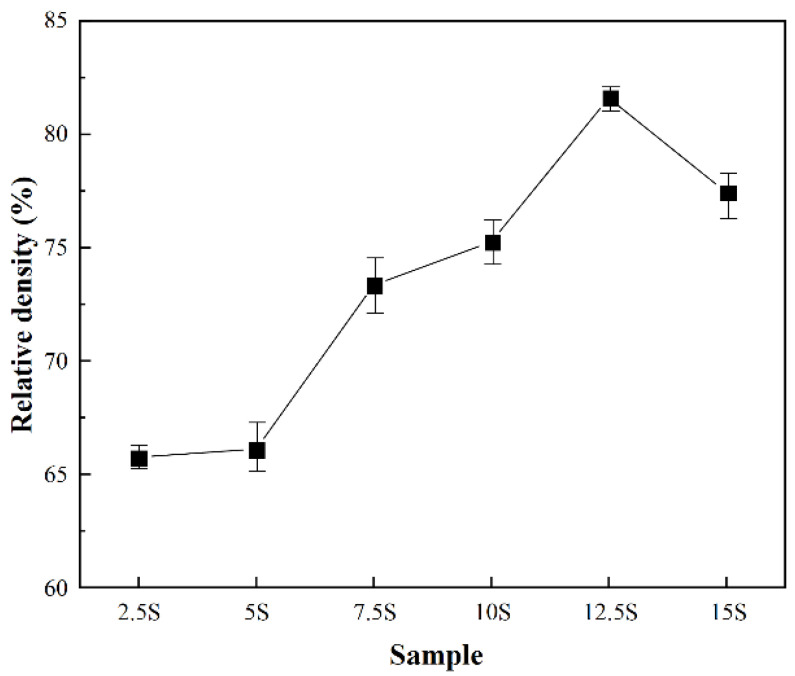
Relative density plots of the LTPO composite samples with different SN to LiTFSI ratios.

Figure 3 shows the variation in the relative density of the LTPO composite pellets with different SN to LiTFSI ratios. As can be seen in Figure 3 and Table 2, all composite pellets exhibited a relative density of 65–85%. Also, the overall relative density increased with the increase in the addition of SN and LiTFSI. This phenomenon would suggest that the presence of SN at the particle interface facilitates particle slip, leading to an increase in the density of the pellet during the compression process. This process makes it possible to create a relative dense structure without the ceramic’s conventional high-temperature sintering.

**Figure 4:**
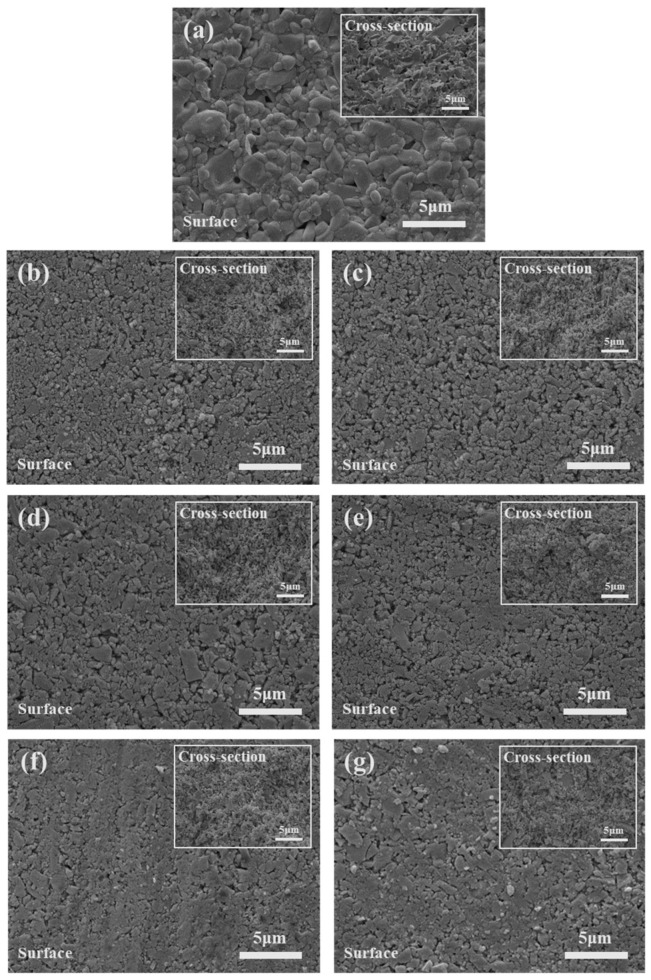
SEM images of the LTPO composite pellets; (**a**) HT, (**b**) 2.5S, (**c**) 5S, (**d**) 7.5S, (**e**) 10S, (**f**) 12.5S, and (**g**) 15S.

Figure 4 presents SEM images of the LTPO composite pellets’ surface and cross-sections with different SN and LiTFSI contents. In the case of the HT sample in Figure 4a, the surface exhibited a compact structure, well-defined grain boundaries, and growth of LTPO particles. In LTPO composite samples, particle growth was not seen, compared with the HT sample. This suggests that SN and LiTFSI assisted in bonding between particles and lead to the dense microstructure of sample. On the other hand, as shown in Figure 4b,c, 2.5S and 5S LTPO composite pellets had relatively more pores compared to other LTPO composite samples. This is well matched with the results of relative density which exhibited a low value of 64% in Figure 3. However, at higher SN and LiTFSI contents, the pores on the surface of the composite pellets disappeared and then the particles were tightly connected with each other, leading to highly dense surface structure. This behavior can be explained by considering that SN existed at the interface of the LTPO particles and improved the physical contact between the ceramic particles.

**Figure 5:**
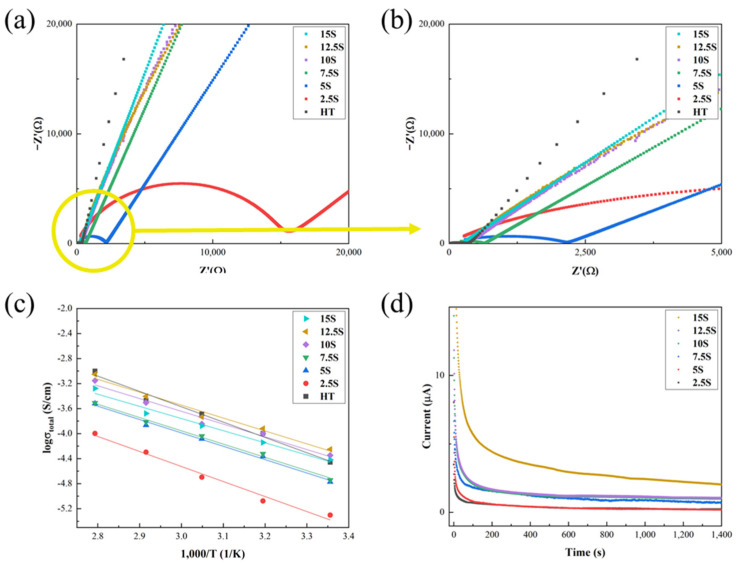
(**a**) Electrochemical impedance spectra, (**b**) magnification of the circle in (**a**), (**c**) Arrhenius plots, and (**d**) direct-current polarization curves of the HT sample and LTPO composite samples.

Figure 5 represents the electrochemical impedance spectra, Arrhenius plots, and direct-current polarization curves of the LTPO composite pellets with different SN and LiTFSI contents. As shown in Figure 5a,b, all samples had their specific semicircle, which represents the total resistance of the electrolyte. The total resistance decreased with increases in the amount of SN and LiTFSI. To calculate the ionic conductivity of the samples, EIS data were fitted using the ZView program. Table 3 represents the values of the electrochemical properties of the LTPO and LTPO composite samples. In the case of the LTPO composite samples, the total ionic conductivity increased with increases in the SN and LiTFSI contents. In addition, the total ionic conductivity (1.93 × 10^−4^ S/cm) of the 12.5S sample was higher than that of the HT sample, although the composite sample had lower relative density compared to the HT sample. The higher ionic conductivity compared to the HT sample observed in the 12.5S sample suggests that the addition of SN and LiTFSI plays a critical role in enhancing the ion transport pathways within the LTPO matrix. Although density is an important factor in ionic conductivity, this study demonstrates that the composite can achieve improved ionic conductivity through the addition of SN and LiTFSI, independent of density. On the other hand, the total ionic conductivity of the 15S sample decreased compared to that of 12.5S sample. This behavior could be explained by considering that there exists some amount of unreacted SN after the maximum (beyond 12.5S). The unreacted SN could act as an insulator blocking ion motion, similar to that observed in the case of an oxide filler [36]. Figure 5c shows the Arrhenius plots of the LTPO composite samples measured in the range of temperature from 25 to 85 °C. As can be seen in Table 3, the activation energy of the LTPO composite sample decreased as the contents of the SN and LiTFSI increased. The overall activation energies of the LTPO composite samples were exhibited to be in the range of 0.197–0.207 eV, which are lower than the HT sample. Figure 5d indicates the direct-current polarization curves of the LTPO composite samples. The LTPO composite samples exhibit relatively low steady-state current, which increased as the contents of the SN increased. The electronic conductivities were obtained using Figure 5d and Equation (1) as shown in Table 3. The electronic conductivities of the LTPO composite samples were in the order of 10^−8^–10^−9^ S/cm at room temperature. These low electronic conductivities could be sufficient to inhibit the formation of lithium dendrites, which is induced by the rapid recombination of electrons and Li^+^ in the Li electrode on the solid electrolyte having the high electronic conductivity [37,38].

**Figure 6:**
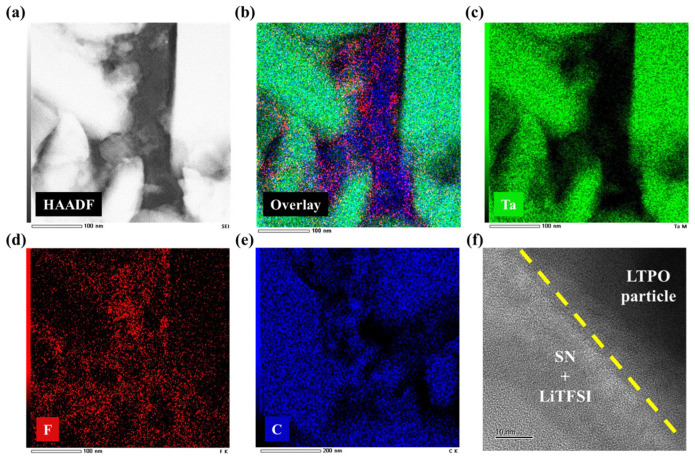
(**a**) HAADF-STEM image with (**b**–**e**) EDS and overlay images, and (**f**) TEM image of a 12.5S sample.

**Figure 7:**
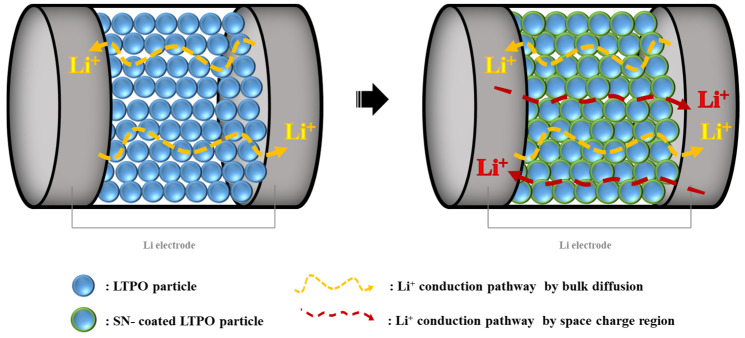
The schematic diagram of the Li^+^ conduction mechanism in the LTPO-based SN and LiTFSI composite solid electrolyte with the Li electrode.

Figure 6 shows a HAADF-STEM image with EDS and TEM images of an LTPO composite sample, respectively. As shown in Figure 6a, there are two distinct areas; the bright area belongs to LTPO particles, and the dark area represents an amorphous phase including the SN and LiTFSI components. As shown in Figure 6b, the overlay image displayed that the interface region mainly contains F and C, which come from SN and LiTFSI compared with the particle part. This microstructure, with particles surrounded by SN and LiTFSI, provides the mechanism for the enhancement of the ionic conductivity of LTPO composite samples aided by SN and LiTFSI. The polar nature of SN may promote better dissociation of lithium salts (LiTFSI), leading to a higher concentration of mobile lithium ions (free charge carrier concentration) in the electrolyte [39]. As a result, the LTPO composite electrolyte mixed with SN and lithium salt exhibits not only bulk diffusion of Li^+^ but also ionic conduction due to the formation of a space charge layer where the concentration of dissociated Li^+^ in LiTFSI increases. This also allows for ionic conduction through the dissociation of Li^+^ and SN [40]. As can be seen in Figure 7, the SN embedded between the LTPO particles facilitates the physical contact between the LTPO particles. This provides ion transport pathways by reducing resistance between the particles, thereby allowing Li^+^ to move between particles more easily. As can be seen in Figure 6c–e, Tantalum is uniformly distributed in LTPO particles, and the element fluorine mainly appeared at the interface between LTPO particles. In contrast, carbon is spread out across all regions including the interface and particle, though it is not a component of the LTPO particle. This could be explained by considering that carbon was deposited onto the LTPO particles during the FIB process for obtaining the TEM sample. The higher magnification TEM image in Figure 6f shows that the LTPO particle is well attached with the SN and LiTFSI layer.

**Figure 8:**
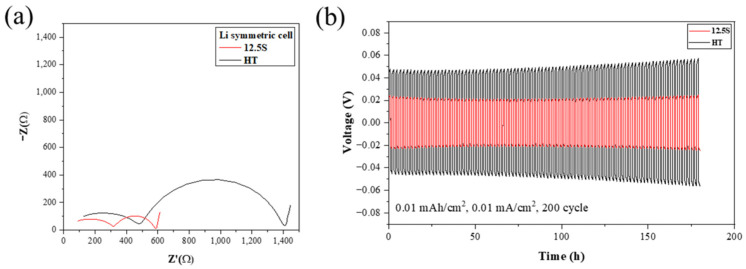
(**a**) Nyquist plots of the Li symmetric cells and (**b**) galvanostatic cycling profile of the Li symmetric cells at 0.01 mA/cm^2^ at RT.

The Li symmetric cell test was carried out for conducting a comparative analysis of the electrochemical properties of lithium symmetric cells with LTPO (HT sample) and LTPO composite (12.5S sample) SEs. Figure 8a presents the Nyquist plots obtained by Li symmetric cell. The first semicircle exhibits the resistance between the bulk and grain boundary, and the next semicircle represents the resistance at the interface between the solid electrolyte and the Li electrode [41]. As shown in Figure 8a, the total resistance of 12.5S is significantly lower than that of HT. At the point of the second semicircle, the LTPO composite of 12.5S had a much lower value compared to the HT sample, indicating that the SN reduced the resistance between the solid electrolyte and the Li electrode. This is attributed to the anchoring effect of SN on the interface between the Li metal and solid electrolyte. SN creates a stable ionic conduction pathway at the interface, reducing the formation of SEI and suppressing direct reactions between the lithium metal and the LTPO composite. Such an anchoring effect effectively lowers interfacial resistance by protecting the lithium electrode from unfavorable reactions. This leads to a reduction in side reactions, which improves the long-term cycling performance and stability of the system. The addition of high concentrations of SN and lithium salt is beneficial in anchoring free SN molecules and in alleviating side reactions between the composite electrolyte and the Li metal [42]. This implies that the LTPO composite samples have low particle interfacial resistance and excellent interfacial contact at the Li electrode, reducing the electrolyte’s total resistance between the interface of the solid electrolyte and the Li metal electrode. Figure 8b presents the galvanostatic cycling profile obtained by Li symmetric cells. Galvanostatic cycle tests were conducted to verify the application of the LTPO composite solid electrolyte in the Li metal battery. In HT, the primary overpotential was 440 mV, which elevated with an increasing number of cycles to 530 mV. It can be seen that the voltage keeps rising as each charge and discharge cycle proceeds, suggesting that the HT sample is unstable with the Li electrode as the electrochemical reaction progresses. In contrast to HT, the voltage change of the 12.5S maintained stability at around 200 mV for all cycles without significant fluctuations. This is likely due to the SN entering the interface of the LTPO, which increases its stability with the Li electrode.

## 4. Conclusions

To summarize, LTPO-based SN and LiTFSI composite solid electrolyte was successfully prepared without the sintering process. The incorporation of SN and LiTFSI did not affect the phase of LTPO, which means that there are no chemical reactions between LTPO and SN–LiTFSI. The obtained LTPO composite had an ionic conductivity of 1.93 × 10^−4^ S/cm with a relative density of 81.1%. This comes from the idea that the SN, formed at the interface between the LTPO particles, plays a role in facilitating slip compaction during the compression process and enables an increase in the ionic conductivity. The LTPO composite also exhibited low values of electronic conductivity, suggesting that the addition of SN could prevent the growth of Li dendrites in the battery-use environment. Furthermore, the LTPO composite exhibited lower interfacial resistance with the Li metal than that of the conventional LTPO solid electrolyte in both the Li symmetric cell test and the galvanostatic cycle test, thereby ensuring electrochemical stability with the Li electrode. On the basis of these results, it was found that the performance of solid electrolytes could be improved by introducing SN and lithium salt into the ceramic solid electrolytes. In addition, the findings demonstrate that the addition of SN could improve the electrochemical performance of solid electrolytes without the high-temperature sintering process.

## Figures and Tables

**Table 1:** Notation of the LTPO-based SN and LiTFSI (SN:LiTFSI = 1:1) composite samples.

Composition (wt%)	Notation
100 LTPO High-temperature sintering (1050 °C—6 h)	HT
97.5 LTPO + 2.5 (SN + LiTFSI)	2.5S
95 LTPO + 5 (SN + LiTFSI)	5S
92.5 LTPO + 7.5 (SN + LiTFSI)	7.5S
90 LTPO + 10 (SN + LiTFSI)	10S
87.5 LTPO + 12.5 (SN + LiTFSI)	12.5S
85 LTPO + 15 (SN + LiTFSI)	15S

**Table 2:** Diameter, thickness, measured density, theoretical density, and relative density of the LTPO composite samples with different SN to LiTFSI ratios.

Sample	Diameter (mm)	Thickness (mm)	Measured Density (g/cm^3^)	Theoretical Density (g/cm^3^)	Relative Density (%)
HT	8.3	0.337	5.471	5.848	93.5
2.5S	10	0.338	3.767	5.746	65.6
5S	10	0.346	3.680	5.644	65.2
7.5S	10	0.308	4.134	5.542	74.6
10S	10	0.315	4.042	5.440	74.3
12.5S	10	0.301	4.244	5.236	81.1
15S	10	0.323	3.942	5.032	78.3

**Table 3:** Total ionic conductivities, activation energies, and electronic conductivities of the LTPO composite samples with different SN to LiTFSI ratios.

Sample	Total Ionic Conductivity at RT (S/cm)	Activation Energy (eV)	Electronic Conductivity at RT (S/cm)
HT	1.83 × 10^−4^	0.211	5.34 × 10^−8^
2.5S	2.86 × 10^−6^	0.207	9.90 × 10^−9^
5S	2.03 × 10^−5^	0.184	7.93 × 10^−9^
7.5S	6.06 × 10^−5^	0.183	2.75 × 10^−8^
10S	9.60 × 10^−5^	0.176	4.01 × 10^−8^
12.5S	1.93 × 10^−4^	0.178	4.01 × 10^−8^
15S	1.83 × 10^−4^	0.167	8.31 × 10^−8^

## Data Availability

Data are contained within the article.
